# Effects of crocin and safranal, saffron constituents, on the formalin-induced orofacial pain in rats

**Published:** 2015

**Authors:** Amir Erfanparast, Esmaeal Tamaddonfard, Mina Taati, Milad Dabbaghi

**Affiliations:** 1 *Department of Basic Sciences, Faculty of Veterinary Medicine, Urmia Universiry, Urmia 57153-1177, Iran *

**Keywords:** *Crocin*, *Safranal*, *Diclofenac*, *Orofacial pain*, *Rats*

## Abstract

**Objective:**

Crocin and safranal are the main components of saffron, and have many biological functions such as anti-inflammatory and antioxidant activities. In the present study, we investigated the effects of crocin, safranal, morphine, diclofenac and naloxone in combined and separately on formalin-induced orofacial pain in rats.

**Materials and Methods:**

Subcutaneous injection of a diluted formalin solution (50 µl, 1.5%) into the upper lip region produced a biphasic pattern of pain response (a neurogenic phase: 0-3 min and an inflammatory phase: 15-33 min). The time each animal spent face rubbing with ipsilateral forepaw was recorded and considered as an index of nociception

**Results:**

Intraperitoneal injections of crocin (12.5 and 25 mg/kg), safranal (0.25 and 0.5 mg/kg), diclofenac (5 and 10 mg/kg) and morphine (1 and 2 mg/kg) suppressed the second phase of pain. The second phase of pain was also reduced when low (ineffective) doses of crocin (6.25 mg/kg) and safranal (0.125 mg/kg) were co-administered with low doses of diclofenac (2.5 mg/kg) and morphine (0.5 mg/kg). The more antinociceptive effects were observed when the medium doses of the above-mentioned chemicals used together. Naloxone prevented morphine-induced antinociception, but did not inhibit the suppressive effects of crocin and safranal. Safranal at a high dose (0.5 mg/kg) suppressed locomotor activity.

**Conclusion:**

The present results showed antinociceptive effects for crocin and safranal in inflammatory pain. Opioid receptors may not be involved in the antinociceptive effect of crocin and safranal. Crocin and safranal increased diclofenac-induced antinociception.

## Introduction

*Crocus sativus L.,* commonly known as saffron, is used in folk medicine for various purposes such as an antispasmodic, nerve sedative, expectorant, eupeptic, anticatarrhal, carminative, diaphoteric, stomachic, aphrodisiac and emmenagogue (Schmidt et al., 2007). Saffron contains carotenoid pigments called tricrocin, bicrocin and crocin, a bitter glycoside called picrocrocin, and the volatile, aromatic substance safranal (Rios et al., 1996 ▶). Crocin, picrocrocin and safranal are responsible for saffron exclusive color, taste and odor, respectively (Melnyk et al., 2010 ▶). Pharmacological studies have demonstrated antiepileptic, anti-oxidative, anti-inflammatory, neuroprotective, memory improvement and anti-diabetic effects for crocin and safranal (Assimopolou et al., 2005 ▶; Nam et al., 2010 ▶; Gadrdoost et al., 2011 ▶; Boskabady et al., 2012 ▶; Tamaddonfard et al., 2012 ▶; Tamaddonfard et al., 2013a, b, c, d). Recent studies have showed anitinociceptive effects for crocin and safranal in various models of pain. Crocin suppressed corneal nociception and formalin-induced pain in rats (Tamaddonfard and Hamzeh-Gooshchi, 2010a ▶, b ▶). Hosseinzadeh and Shariaty (2007) ▶ showed antinociceptive and anti-inflammatory properties for safranal in the formalin, writhing and hot plate tests of nociception in mice. Moreover, crocin attenuated cold and mechanical allodynia induced by intraplantar injection of carrageenan in rats (Tamaddonfard et al., 2013c ▶). Amin and Hosseinzadeh (2012) ▶ showed that safranal decreased the intensity of pain responses in chronic constriction injury model of neuropathic pain in rats. In addition, another study reported that neuropathic pain responses induced by sciatic nerve crush injury were suppressed by safranal (Tamaddonfard et al., 2013d).

The orofacial region is one of the most densely innervated (by the trigeminal nerve) areas of the body, which focuses some of the most acute and chronic pains (Sessle, 2011 ▶). Pain information from the oral and facial structures including upper lip, mucosa of nose, lower lip and chin, cornea, conjunctiva are transmitted through maxillary, mandibular and ophthalmic divisions of trigeminal nerve to the trigeminal brainstem sensory nuclear complex where they relay to higher levels of the brain (Guy et al., 2005 ▶). Opioid receptors agonist, morphine, and cyclooxygenase pathways product inhibitors (NSAIDs: non-steroidal anti-inflammatory drugs) are the most commonly used drugs in management of orofacial pain (Ganzberg, 2010 ▶; Sarlani et al., 2005 ▶). Due to the side effects of NSAIDs (Suleyman et al., 2007 ▶) and morphine (Benyamin et al., 2008 ▶), there is a great interest in natural compounds, such as dietary supplements and herbal remedies, which have been used for centuries to reduce pain and inflammation (Marron et al., 2010 ▶). 

There are some reports that the anatomical and physiological consequences of nerve injuries of the trigeminal system differ from those seen after peripheral nerve injury (Bongenhielm et al., 1999 ▶; Tal and Devor, 1992 ▶). Although some scholars have showed anitinociceptive effects for crocin and safranal in various models of pain (Tamaddonfard and Hamzeh-Gooshchi, 2010a ▶, b ▶; Amin and Hosseinzadeh, 2012 ▶), effects of crocin and safranal on the orofacial pain in rats have not been investigated yet. The above considerations raised our interest on the issue of testing the effect of crocin and safranal in orofacial pain. To clarify the possible mechanisms of action , we used morphine, naloxone and diclofenac in combined treatments with crocin and diclofenac. 

The orofacial formalin test was introduced by Clavelou et al. (1989) ▶ and completed by Clavelou et al. (1995). This model of orofacial pain has been frequently used with success in the study of the pain mechanisms originating from orofacial region (Chichorro et al., 2004 ▶; Duale et al., 2007 ▶; Burgos et al., 2010 ▶; Erfanparast et al., 2014 ▶). 

## Materials and Methods

**Animals**

Healthy adult male Wistar rats, weighing 230–260 g were used in this study. The animals were provided from rat house of Laboratory of Physiology of Faculty of Veterinary Medicine of Urmia University. Rats were maintained in groups of six per cage in a 12 h light-dark cycle (light on at 07:00 h) at a controlled ambient temperature (22 ± 0.5 ° C) with ad libitum food and water. Six rats were used in each experiment. All experiments were performed between 12:00 h and 17: 00 h. All research and animal care procedures were approved by the Veterinary Ethics Committee of the Faculty of Veterinary Medicine of Urmia University, and were performed in accordance with the National Institutes of Health Guide for Care and Use of Laboratory Animals.

**Drugs**

Drugs used in this study included crocin (Fluka, Germany), safranal, diclofenac sodium, naloxone hydrochloride (Sigma-Aldrich, USA) and morphine sulfate (Temad, Iran). Crocin, diclofenac, morphine and naloxone were dissolved in normal saline. Safranal was dissolved in liquid paraffin (Amin and Hosseinzadeh, 2012 ▶; Tamaddonfard et al., 2013c ▶). 

**Treatment groups**

In the present study, 156 male Wistar rats were divided into 26 groups with six rats in each group as follows: Groups 1 and 2 received normal saline and paraffin, respectively. Groups 3, 4, 5, 6, 7, 8, 9, 10, 11, 12, 13 and 14 received crocin at doses of 6.25, 12.5 and 25 mg/kg, safranal at doses of 0.125, 0.25 and 0.5 mg/kg, morphine at doses of 0.5, 1 and 2 mg/kg and diclofenac at doses of 2.5, 5 and 10 mg/kg, respectively. Groups 15, 16, 17, 18 treated with crocin (6.25 mg/kg) plus morphine (0.5 mg/kg), safranal (0.125 mg/kg) plus morphine (0.5 mg/kg), crocin (12.5 mg/kg) plus morphine (1 mg/kg) and safranal (0.25 mg/kg) plus morphine (1 mg/kg), respectively. Groups 19, 20, 21 and 22 received crocin (6.25 mg/kg) plus diclofenac (2.5 mg/kg), safranal (0.125 mg/kg) plus diclofenac (2.5 mg/kg), crocin (12.5 mg/kg) plus diclofenac (5 mg/kg) and safranal (0.25 mg/kg) plus diclofenac (5 mg/kg), respectively. Groups 23, 24, 25 and 26 treated with naloxone (2 mg/kg), naloxone (2 mg/kg) plus morphine (2 mg/kg), naloxone (2 mg/kg) plus crocin (25 mg/kg) and naloxone (2 mg/kg) plus safranal (0.5 mg/kg). Crocin, safranal, morphine, naloxone and diclofenac were intraperitoneally injected before subcutaneous injection of formalin.

**Formalin-induced orofacial pain**

Formalin-induced orofacial pain was performed according to the method described by Raboisson and Dallel (2004) ▶ and Tamaddonfard et al. (2011) ▶. Each rat was placed in plexiglass observation chamber (30 cm × 30 cm × 30 cm) with a mirror mounted at 45° beneath the floor to allow an unobstructed view of the orofacial region. After a 30-min adaptation period, 50 µl of 1.5% diluted formalin solution was subcutaneously injected into the left side of upper lip just lateral to the nose using a 30-gauge injection needle. Immediately following formalin injection, the rat was returned into the observation chamber. The time each animal spent face rubbing with ipsilateral forepaw was recorded (using a stopwatch), in consecutive 3-min blocks over a period of 45 min, and was considered as an index of nociception. Subcutaneous injection of formalin (0.2–10%) into the upper lip induced a stereotyped response characterized by two well distinct phases (Clavelou et al., 1989 ▶; Clavelou et al., 1995 ▶; Raboisson and Dallel, 2004 ▶; Tamaddonfard et al., 2011 ▶). Data collected between 0 and 3 min post-formalin injection represented first (neurogenic) phase and data collected between 15 and 33 min after injection of formalin represented second (inflammatory) phase as described by Tamaddonfard et al. (2011) ▶. All the observers were blinded to the protocol of the study. 

**Locomotor activity**

Locomotor activity was assessed in an open-field test as described previously (Markowska and Lukaszewska, 1981 ▶). The apparatus consisted of a wooden box measuring 120 cm × 120 cm × 50 cm. The floor of the arena was divided into 16 equal squares. To monitor the activity, animals were removed from the home cage and placed directly into one corner of the open field apparatus. The number of squares crossed with all paws (crossings) and the number of rearing were counted in a 5-min session. 

**Study protocol**

Before rats were pain tested, they were placed in the formalin test chamber for 30 min on three successive days to minimize stress-activated pain suppressive mechanisms (Abbot and Bonder, 1997 ▶). Crocin and safranal were intraperitoneally injected 30 min before orofacial pain induction. Diclofenac was injected 25 min before subcutaneous injection of formalin into lip region. Naloxone and morphine were intraperitoneally injected 35 and 25 min before induction of orofacial pain, respectively. This time schedule was also applied for open-field test. The intraperitoneal injection volume was 1 ml/kg. The doses of crocin, safranal, morphine, diclofenac and naloxone were designed according to previous studies, which were at 12.5-100 mg/kg, 0.25-2 mg/kg, and 2-10 mg/kg, 2.5-10 mg/kg and 1-2 mg/kg, respectively (Farshid et al., 2010 ▶; Burgos et al., 2010 ▶; Boskabady et al., 2012 ▶; Tamaddonfard et al., 2013c ▶). 

**Statistical analysis**

The data obtained from subcutaneous injection of formalin into upper lip region were analyzed using two-way analysis of variance (ANOVA) followed by Duncanʼs test. Comparison among data obtained from first and second phases of formalin-induced orofacial pain were performed by one-way analysis of variance (ANOVA) followed by Duncanʼs test. Significance at p<0.05 and p<0.01 have been given receptive in the figures.

## Results

**Figure 1:**
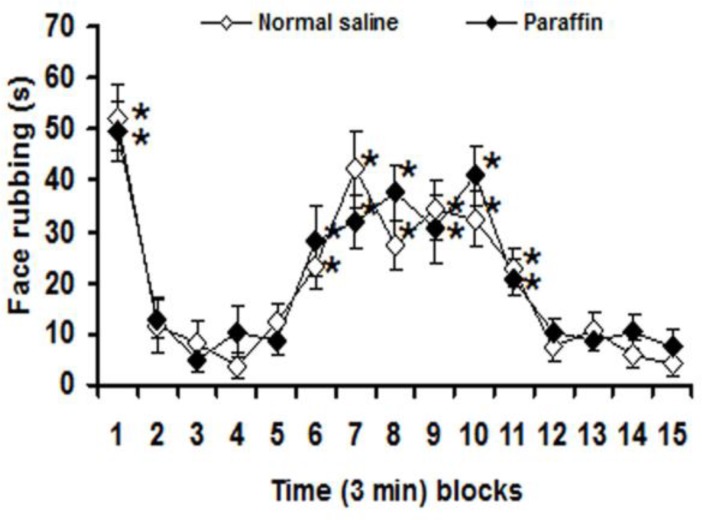
Three-min blocks of face rubbing induced by injection of formalin into the upper lip after intraperitoneal normal saline and liquid paraffin. Values are expressed as the mean ± SEM. (n = 6 rats in each group). * p<0.05 compared with other 3-min blocks

With no significant differences between normal saline and paraffin treated groups on 3-min blocks nociceptive response, significant (p<0.05) differences in face rubbing were observed among first, 6th–11th and other 3-min blocks after subcutaneous injection of formalin in both normal saline and paraffin treated groups (Figure 1). 

**Figure 2:**
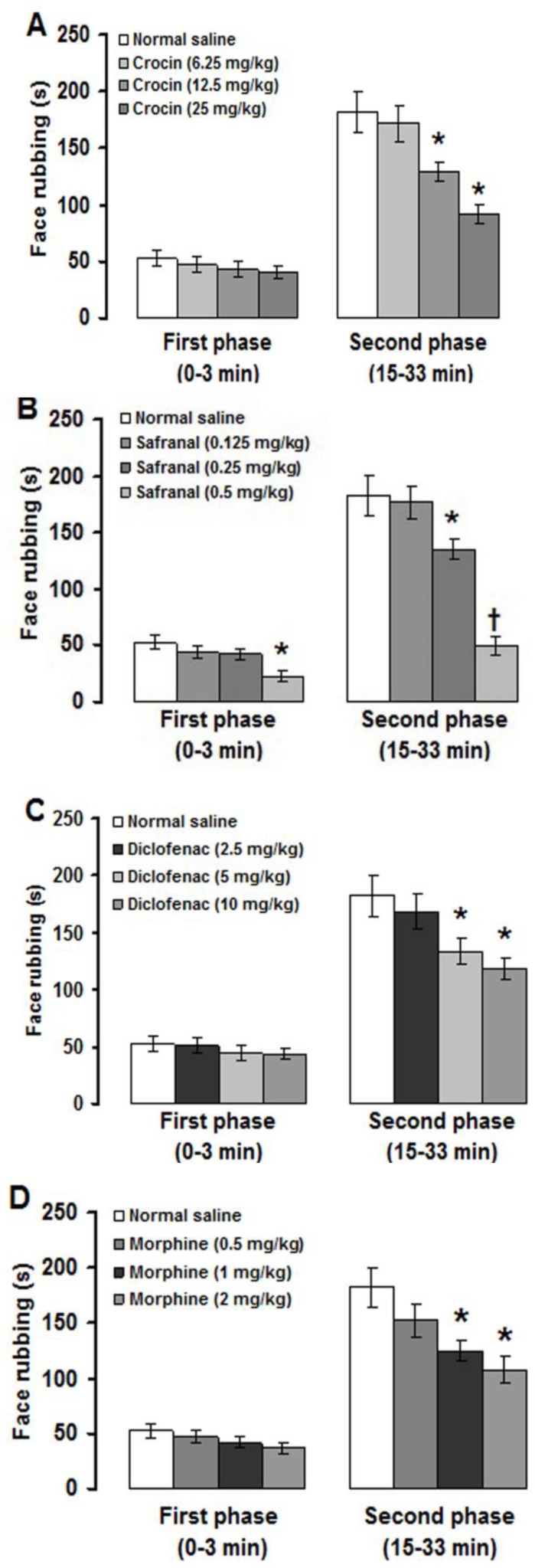
Effects of intraperitoneal injections of crocin (A), safranal (B), diclofenac (C) and morphine (D) on formalin-induced orofacial pain. Values are expressed as the mean ± SEM. (n = 6 rats in each group). * p<0.05, ^†^ p<0.01 compared with normal saline treated group

This means that intraperitoneal injection of paraffin had no effects on pain severity. Therefore, the obtained data from experimental groups were compared with intraperitoneal normal saline treated group. Crocin, safranal, diclofenac and morphine at doses of 6.25 mg/kg, 0.125 mg/kg, 2.5 mg/kg and 0.5 mg/kg, respectively, did not affect the first and second phases of formalin-induced orofacial pain (Figures 2A, 2B, 2C, 2D). Crocin at doses of 12.5 and 25 mg/kg, safranal at doses of 0.25 and 0.5 mg/kg, diclofenac at doses of 5 and 10 mg/kg and morphine at doses of 1 and 2 mg/kg significantly (p<0.05) reduced the second phase of face rubbing induced by upper lip injection of formalin (Figures 2A, 2B, 2C, 2D). In addition, safranal at a dose of 0.5 mg/kg also significantly (p<0.01) attenuated the first phase of pain (Figure 2B). 

**Figure 3:**
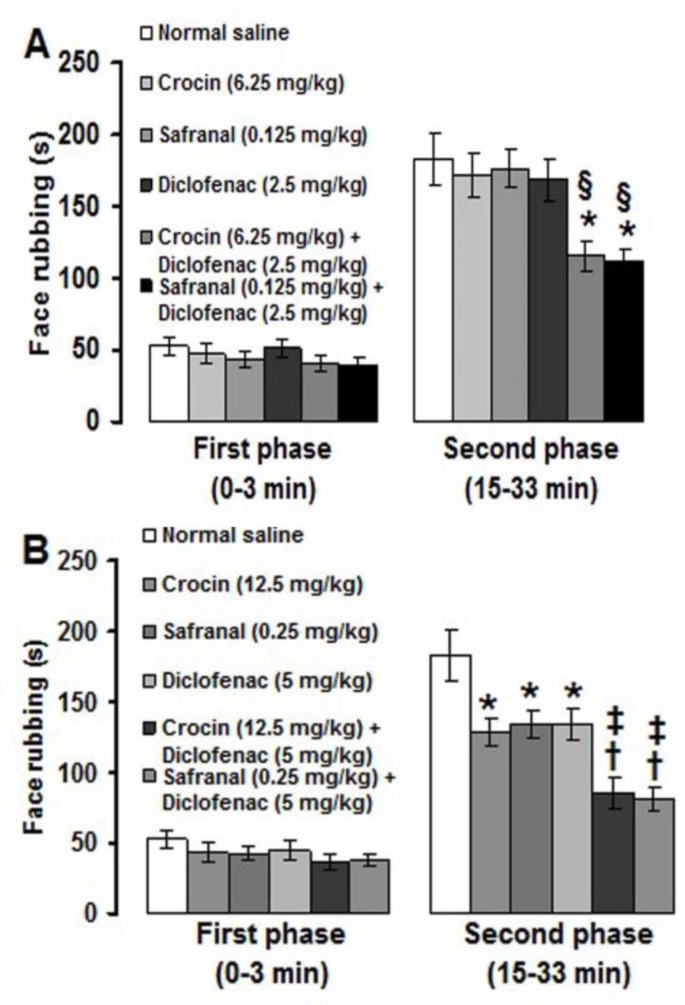
Effects of combination treatments with sub-analgesic (A) and analgesic (B) doses of crocin and safranal with diclofenac on the orofacial pain induced by formalin. Values are expressed as the mean ± SEM. (n = 6 rats in each group). * p<0.05, ^†^ p<0.01 compared with normal saline treated group. § p<0.05 compared with crocin (6.25 mg/kg) and safranal (0.125 mg/kg) treated groups. ^‡^ p<0.05 compared with crocin (12.5 mg/kg) and safranal (0.25 mg/kg) treated groups

When low (sub-analgesic) doses of crocin (6.25 mg/kg) and safranal (0.125) were co-administered with a low dose of diclofenac (0.5 mg/kg), the second phase of formalin-induced pain was significantly (p<0.05) suppressed (Figure 3A). Medium (analgesic) doses of crocin (12.5 mg/kg) and safranal (0.25 mg/kg) produced significantly (p<0.01) more suppressive effects on the second phase of pain when used together with a medium dose (5 mg/kg) of diclofenac (Figure 3B).

**Figure 4:**
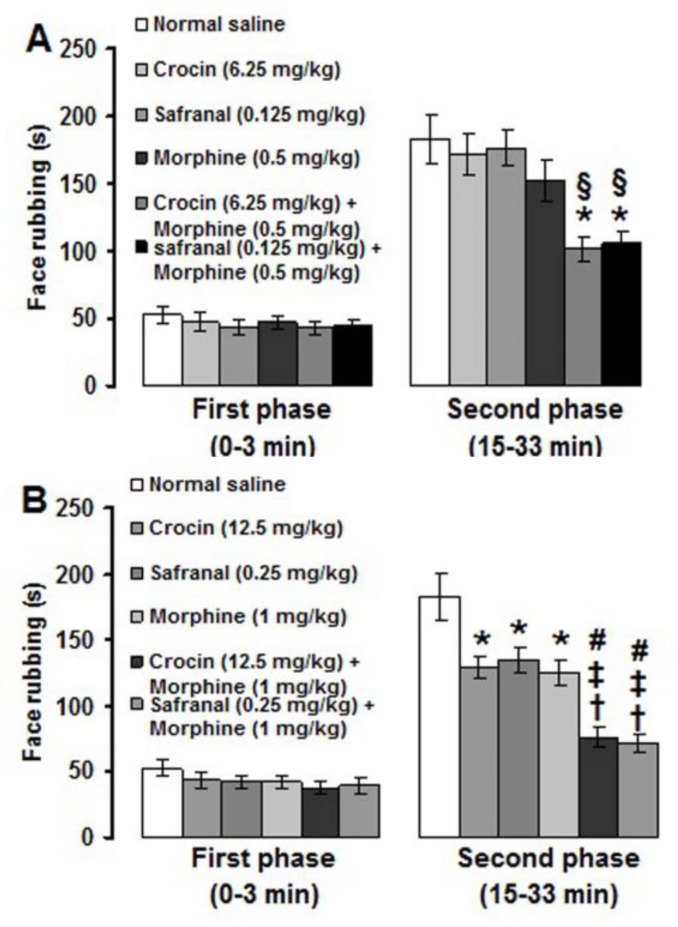
Effects of sub-analgesic (A) and analgesic (B) doses of crocin and safranal incombination treatments with morphine on formalin-induced orofacial pain. Values are expressed as the mean ± SEM. (n = 6 rats in each group). * p<0.05, ^†^ p<0.01 compared with normal saline treated group. § p<0.05 compared with crocin (6.25 mg/kg) and safranal (0.125 mg/kg) treated groups. ^‡^ p<0.05 compared with crocin (12.5 mg/kg) and safranal (0.25 mg/kg) treated groups. # p<0.05 compared with crocin (6.25 mg/kg) plus morphine (0.5 mg/kg) and safranal (0.125 mg/kg) plus morphine (0.5 mg/kg) treated groups

Co-administrations of low (sub-analgesic) doses of crocin (6.25 mg/kg) and safranal (0.125 mg/kg) with a low dose of morphine (0.5 mg/kg) significantly (p<0.05) produced antinociceptive effects in the second phase of formalin-induced pain (Figure 4A). Medium (analgesic) doses of crocin (12.5 mg/kg) and safranal (0.25 mg/kg) significantly (p<0.01) produced suppressive effects on the second phase of pain when used together with a medium dose (1 mg/kg) of morphine (Figure 4B). The more suppressive effects were obtained in medium dose combination treatments than those observed in low dose combination treatments (p<0.05) (Figures 4A, 4B).

**Figure 5:**
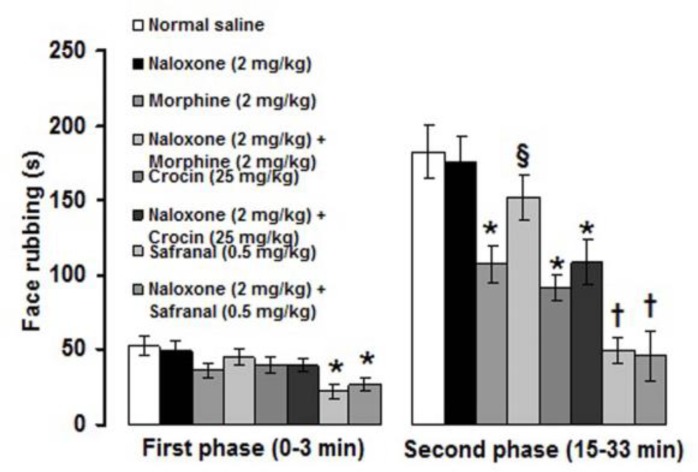
Effects of naloxone on the antinociceptive effects induced by morphine, crocin and safranal in the formalin-induced orofacial pain. Values are expressed as the mean±SEM. (n=6 rats in each group). * p<0.05, ^†^ p<0.01 compared with normal saline treated group. § p<0.05 compared with morphine (2 mg/kg) treated group

**Figure 6:**
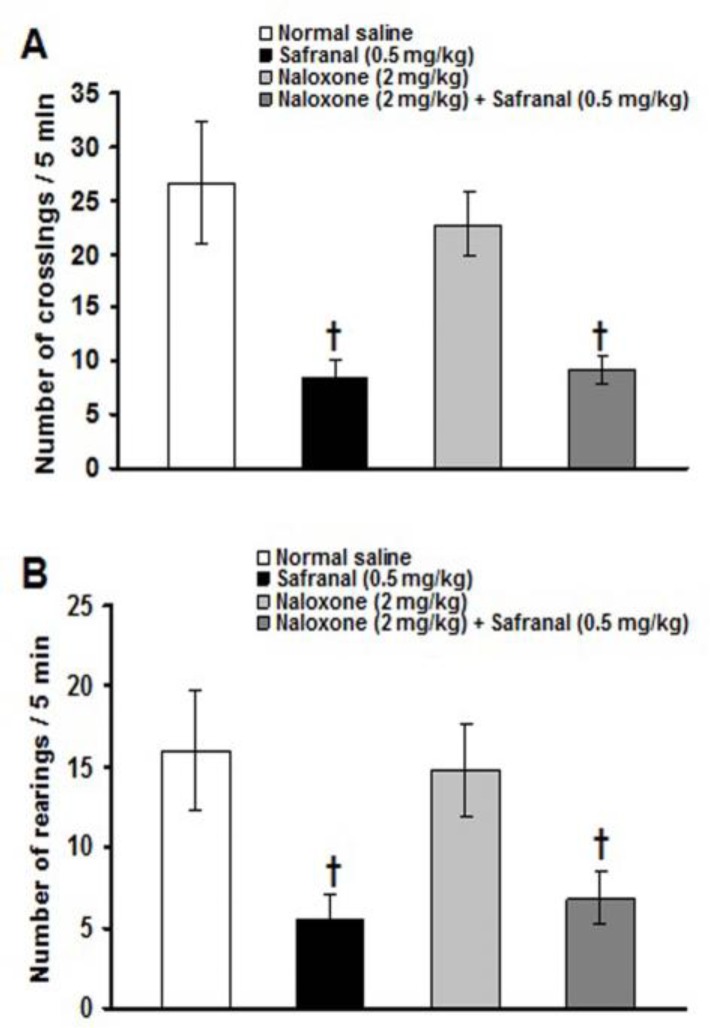
Effects of safranal, naloxone and naloxone plus safranal on the numbers of crossing (A) and rearing (B) in the activity box. Values are expressed as the mean ± SEM. (n = 6 rats in each group). ^†^ p<0.01 compared with naloxone and normal saline treated groups

Naloxone (2 mg/kg) alone did not change the severity of formalin-induced orofacial pain (Figure 5). Morphine (2 mg/kg)-induced antinociception was significantly (p<0.05) inhibited with 2 mg/kg of naloxone (Figure 5). Naloxone (2 mg/kg) did not inhibit the antinociceptive effects induced by 25 mg/kg of crocin and 0.5 mg/kg of safranal (Figure 5). The number of crossing and rearing were 26.7 ± 5.7 and 16 ± 3.8, respectively, in intraperitoneal normal saline treated group. All the separately and combined treatments, did not affect the number of crossing and rearing (data not shown), except of those obtained from 0.5 mg/kg of safranal and 2 mg/kg of naloxone plus 0.5 mg/kg of safranal. Safranal (0.5 mg/kg) significantly (p<0.01) decreased the numbers of crossing and rearing and naloxone did not prevent safranal-induced suppression of locomotion (Figures 6A, 6B).

## Discussion

The present study showed that subcutaneous injection of formalin (1.5%) into the upper lip produced a distinct biphasic pattern of face rubbing. The first (neurogenic) phase began immediately after formalin injection and declined in approximately six min, while the second (inflammatory) phase began about 15 min after formalin injection and lasted about 18 min and declined to the end of recording period (45 min). This nociceptive behavior is in agreement with other investigations (Clavelou et al., 1995 ▶; Tamaddonfard et al., 2011 ▶).

In this study, crocin and safranal, with no effect on the first phase, suppressed the second phase of formalin-induced orofacial pain. However, safranal at a high dose suppressed the first phase and produced a more suppressive effect on the second phase of formalin-induced orofacial pain. The first phase in turn may be attributed to a direct algogenic effect of formalin on the nociceptors and the second phase to release of local inflammatory mediators responsible for sensitization of primary and spinal sensory neurons and subsequent signal transduction into the brain (Tjolsen et al., 1992 ▶; Porro and Cavazzuti, 1993 ▶; Raboisson and Dallel, 2004 ▶). There are not any reports showing the effects of crocin and safranal on orofacial pain induced by formalin. Crocin dose-dependently reduced licking and biting response induced by intraperitoneal injection of formalin in rats (Tamaddonfard and Hamzeh-Gooshchi, 2010a ▶). Moreover, systemic and intracerebroventricular (i.c.v.) injections of crocin suppressed pain response in acute trigeminal pain induced by local corneal surface application of hypertonic saline in rats (Tamaddonfard and Hamzeh-Gooshchi, 2010b ▶). Safranal produced antinociceptive effects in paw formalin, writhing and hot plate tests in mice (Hosseinzadeh and Shariati, 2007 ▶). In addition, cold allodynia, mechanical allodynia and mechanical hyperalgesia produced by intraplantar injection of carrageenan were suppressed with intraperitoneal injections of crocin and safranal (Tamaddonfard et al., 2013c ▶). 

In chronic constriction injury model of neuropathic pain in rats, intraperitoneal injection of safranal, but not crocin, attenuated cold and mechanical allodynia as well as thermal hyperalgesia (Amin and Hosseinzadeh, 2012 ▶). Moreover, safranal suppressed sciatic nerve crush injury-induced neuropathic pain signs in rats (Tamaddonfard et al., 2014 ▶). The results presented here are the first report showing analgesic effects for crocin and safranal in inflammatory pain arising from orofacial structures. 

The results of this study showed that diclofenac suppressed the second phase of formalin-induced orofacial pain. The second phase of formalin-induced pain was also suppressed with co-administrations of sub-analgesic and analgesic doses of crocin and safranal with diclofenac. It is known that diclofenac, as other non-selective NSAIDS, is able to impair prostaglandin synthesis by the inhibition of the cyclooxygenase isozymes COX-1 and COX-2 in both, the injured tissues and the central nervous system (Vane and Botting, 1999 ▶). Some researchers have reported the involvement of COX in formalin-induced orofacial pain in mice and rats (Chichorro et al., 2004 ▶; Miranda et al., 2011 ▶). Local peripheral and intraperitoneal injections of ketorolac and diclofenac, with no effect on first phase, attenuated the second phase of orofacial formalin pain in rats (Padi et al., 2006 ▶; Erfanparast et al., 2014 ▶). Diclofenac was also used as reference drugs in studying the anti-inflammatory and analgesic mechanisms of drugs and plant extracts (Farshid et al., 2010 ▶; Torpe et al., 2011 ▶; Tamaddonfard et al., 2013c ▶, e ▶). Some interactions exist among COX, crocin and safranal. In Frond’s Complete Adjuvant (FCA) model of arthritis in rats, crocin produced better effects than ibuprofen (a NSAID) in reducing paw swelling and serum concentrations of COX-2 and PGE2 (Hemshekhar et al., 2012 ▶). Xu et al. found that oral administration (7 days) of crocin (25 and 50 mg/kg) dose-dependently suppressed both phases (3 and 6 h) of carrageenan induced paw edema in rats. They also showed that crocin inhibited production of prostsglandin E2 in rat paw tissues and LPS-challenged RAW 264.7 cells (Xu et al., 2009 ▶). In addition, Tamaddonfard et al. (2013c) ▶ reported that the anti-inflammatory and antinociceptive effects produced by crocin and safranal were the same as the diclofenac did in carrageenan model of inflammatory pain in rats. 

In the present study, co-administrations of sub-analgesic and analgesic doses of crocin and safranal with morphine suppressed the second phase of formalin-induced orofacial pain. Moreover, morphine-, but not crocin- and safranal-induced antinociception, was inhibited by naloxone pretreatment. These findings indicate that crocin and safranal can use as adjuvant with morphine in reducing pain. In addition, opioid receptors are not involved in crocin- and safranal-induced antinociception. Morphine and naloxone frequently used to explore the role of endogenous opioid analgesic system in peripheral, spinal and supra-spinal trigeminal pain mechanisms (Luccarini et al., 1995 ▶; Eisenberg et al., 1996 ▶; Grabow and Dougherty, 2001 ▶; Tamaddonfard et al., 2011 ▶). Crocin exerted its antinociceptive effects through a naloxone-insensitive mechanism in acute trigeminal and inflammatory models of pain in rats (Tamaddonfard and Hamzeh-Gooshchi, 2010a ▶, b ▶). In the hot plate, writhing and formalin tests of nociception in mice, safranal through a naloxone-independent mechanism produced antinociceptive effects (Hosseinzadeh and Shariati, 2007 ▶). 

 Our findings showed that safranal at a high dose of 0.5 mg/kg suppressed locomotor activity in an open-field test. Locomotor activity was also suppressed with 2 mg/kg of naloxone plus 0.5 mg/kg of safranal. These mean that locomotor activity lowering effect of safranal (at a high dose) may interact with its antinociceptive effects observed on the first and second phases of pain, and this effect was not inhibited by opioid receptor antagonist, naloxone. It has been reported that safranal produces hypnotic effect with increasing the sleeping time induced by pentobarbital in mice (Hosseinzadeh and Noraei, 2009 ▶). Moreover, in a neuropathic pain model induced by chronic constriction injury, intraperitoneal injection of safranal at doses of 0.05 and 0.1 mg/kg for a period of seven days produced sedative effect by decreasing locomotor activity in an open field test in rats (Amin and Hosseinzadeh, 2012 ▶). In the present study, crocin at doses of 6.25, 12.5 and 25 mg/kg did not change locomotor activity. Indeed, crocin at a high dose of 400 mg/kg reduced crossing, sniffing, rearing and grooming in an open field test (Tamaddonfard and Hamzeh-Gooshchi, 2010a ▶). In our study, the used doses of morphine (0.5, 1 and 2 mg/kg), naloxone (2 mg/kg) and diclofenac (2.5, 5 and 10 mg/kg) failed to affect locomotor activity. Cristiano et al. (2008) ▶ reported that intraperitoneal injections of 6 mg/kg of morphine and 20 mg/kg of diclofenac produced no sedative effect in rotarod and spontaneous locomotor tests. The experimental and clinical uses of combinations of analgesic agents have increased significantly in the last few years. The purpose is to associate two or more drugs with different mechanisms of action in hope of achieving a synergistic interaction that yields a sufficient analgesic effect with low doses of each agent, therefore, reducing the intensity and incidence of untoward effects (Curatolo et al., 2002 ▶). In this context, the use of opioid analgesics is associated with a number of adverse effects such as dizziness, sedation, constipation and respiratory depression (Benyamin et al. 2008 ▶). On the other hand, the administrations of the high dose of non-steroidal anti-inflammatory drugs produce several adverse reactions, primarily gastrointestinal toxicity such as hemorrhages and ulcerations (Wolf et al., 1999 ▶). 

In conclusion, our results showed antinociceptive effects for crocin, safranal, morphine and diclofenac. Sub-analgesic doses of crocin and safranal produced antinociceptive effects when concurrently used with sub-analgesic doses of morphine and diclofenac. By increasing the doses of crocin and safranal to the effective doses, the antinociceptive effects were more documented. In addition, opioid receptor may have not a role in antinociceptive effects induced by crocin and safranal. Crocin and safranal increased diclofenac-induced antinociception.
